# Longitudinal neurological analysis of moderate and severe pediatric cerebral visual impairment

**DOI:** 10.3389/fnhum.2022.772353

**Published:** 2022-08-16

**Authors:** Andres Jimenez-Gomez, Kristen S. Fisher, Kevin X. Zhang, Chunyan Liu, Qin Sun, Veeral S. Shah

**Affiliations:** ^1^Neuroscience Center, Joe DiMaggio Children’s Hospital, Hollywood, FL, United States; ^2^Division of Pediatric Neurology, Cincinnati Children’s Hospital Medical Center, Cincinnati, OH, United States; ^3^Department of Pediatrics, University of Cincinnati College of Medicine, Cincinnati, OH, United States; ^4^Division of Pediatric Ophthalmology, Cincinnati Children’s Hospital Medical Center, Cincinnati, OH, United States; ^5^Medical Scientist Training Program, University of Cincinnati College of Medicine, Cincinnati, OH, United States; ^6^Division of Biostatistics and Epidemiology, Cincinnati Children’s Hospital Medical Center, Cincinnati, OH, United States; ^7^Department of Ophthalmology, University of Cincinnati, Cincinnati, OH, United States; ^8^Baylor College of Medicine, Cullen Eye Institute, Houston, TX, United States; ^9^Department of Ophthalmology, Texas Children’s Hospital, Houston, TX, United States

**Keywords:** cerebral vision impairment, cortical visual impairment, brain based visual impairment, epilepsy, cerebral palsy, prematurity, cortical blindness

## Abstract

**Introduction:**

Cerebral visual impairment (CVI) results from damage to cerebral visual processing structures. It is the most common cause of pediatric visual impairment in developed countries and rising in prevalence in developing nations. There is currently limited understanding on how neurologic, developmental, and ophthalmic factors predict outcome for pediatric CVI.

**Method:**

A retrospective manual chart review of pediatric CVI patients seen at the tertiary pediatric hospital neurology and neuro-ophthalmology service between 2010 and 2019 was conducted. Patients were stratified into severity groups (based on a custom CVI grading score), and followed over time to identify outcome predictors. Collected baseline characteristics included perinatal, genetic, developmental, and neurologic history, along with neuroimaging and fundoscopic findings on examination. Longitudinal data collected included age, seizure control, and type of therapy received. Linear mixed-effect models were used for longitudinal CVI grade outcome analysis.

**Results:**

A total of 249 individuals spanning 779 patient visits were identified. Mean age at diagnosis was 18.8 ± 16.8 months (2–108 months). About 64.3% were born at term age. Perinatal history revealed hypoxic ischemic encephalopathy (HIE) in 16.5%, intraventricular hemorrhage (IVH) in 11.6%, and seizures in 21.7%. At presentation, 60.3% had a diagnosis of cerebral palsy and 84.7% had developmental delay. Among all subjects, 78.6% had epilepsy; 33.8% had an epileptic encephalopathy, with spasms/hypsarrhythmia being most common. Abnormal neuroimaging was present in 93.8%. Genetic anomalies were present in 26.9%. Baseline visual examination revealed no blink-to-light (BTL) in 24.5%; only BTL in 34.5%, fixation/tracking in 26.5%, and optokinetic drum follow in 14.4%. Longitudinal data analysis showed that perinatal history of HIE, a positive epilepsy history, using multiple (≥3) epilepsy medications, cerebral palsy, and abnormal fundoscopic findings were all negatively associated with CVI grade change over time. After controlling for significant confounders, receiving any type of therapy [early childhood intervention (ECI), physical and occupational therapy (PT/OT), refractive error correction or glasses] was significantly associated with longitudinal improvement in CVI grade compared to patients who did not receive any therapy, with glasses yielding the largest benefit.

**Conclusion:**

This study offers extensive insights into neurologic, developmental and ophthalmologic features in patients with moderate to severe CVI. In concordance with previous findings, aspects of perinatal history and epilepsy/seizure control may help inform severity and prognosis in the general neurology or ophthalmology clinic. Conversely, these aspects, as well as genetic and specific epilepsy traits may alert vision health care providers in the clinic to pursue visual evaluation in at-risk individuals. Longitudinal follow-up of CVI patients showed that interventional therapies demonstrated vision function improvement greater than no therapy and maturational development.

## Introduction

Cerebral visual impairment (CVI) is a result of damage or maldevelopment of the visual processing centers of the brain. Despite being the leading cause of profound pediatric vision loss in developing nations, there has been a global lack of consensus on the terminology, clinical assessment, recognition of diagnosis, as well as overall management of CVI (Sakki et al., 2018; Kran et al., 2019; Ortibus et al., 2019). This complexity primarily arises in that CVI is not a single entity, but heterogeneous in nature with multiple neurological etiologies, presentations, and severity of impairment (Good et al., 1994; Fazzi et al., 2007; Philip and Dutton, 2014).

Cerebral visual impairment can be defined as visual impairment due to injury of the retro-chiasmal or post-geniculate visual processing pathways that are not attributable to any anterior visual pathway dysfunction (Sakki et al., 2018). Underlying etiologies of CVI can be congenital or acquired, and can develop temporally during the prenatal, perinatal, and postnatal stages of human development (Dutton et al., 2006; Khetpal and Donahue, 2007). Much of the current CVI literature has been focused on terminology, screening for early recognition, and developing an algorithm and consensus of CVI clinical assessment, which commonly necessitates a multidisciplinary approach (Bennett et al., 2019; Kran et al., 2019; Ortibus et al., 2019; Ben Itzhak et al., 2020; Chandna et al., 2021).

Recently, there has been further consideration of a CVI patient’s overall clinical presentation, evaluation, and optimal management (Ortibus et al., 2019; Ben Itzhak et al., 2020). A vast majority of CVI children have comorbid neurological disorders and deficits including neurodevelopmental disabilities, epilepsy/seizures, cerebral palsy, hydrocephalus, genetic changes, periventricular white matter disease, and/or anterior visual pathway diseases (Good et al., 1994; Fazzi et al., 2007; Philip and Dutton, 2014). CVI commonly co-presents with a number of ocular abnormalities of refractive error, strabismus, ocular dysmotility, and optic nerve atrophy (Huo et al., 1999; Hoyt, 2003; Good et al., 1994; Handa et al., 2018). Unrecognized moderate-to-severe anterior visual pathway disease can either mask or compound CVI visual behavior and profoundly impact the clinical approach to fully assessing CVI (Ortibus et al., 2019).

Based on the above, we hypothesized that investigating both neurologic and neuro-ophthalmic features of pediatric CVI patients at presentation and longitudinal follow-up may reveal clinical associations that would assist in subcategorizing this heterogeneous disorder and help optimize management. The primary aim of this pediatric retrospective study was to characterize CVI vision outcomes in children presenting at both neurology and neuro-ophthalmology clinics. Data was collected via manual record review with particular focus on neurologic/neuro-ophthalmologic features of each patient with regards to vision assessment. Secondary aims included investigating longitudinal data in terms of neurologic/neuro-ophthalmologic management, vision development, and identifying common clinical and paraclinical risk factors in CVI. We intend the data in this study to help inform general pediatric, ophthalmology, and neurology practices in electing for early referral of similar patients for neuro-ophthalmic evaluation.

## Materials and methods

The study was performed in compliance with all national and institutional regulations. It was reviewed and approved by the Baylor College of Medicine Institutional Review Board (H-38264). A single-center, retrospective cohort study was designed in a large referral pediatric center (Texas Children’s Hospital). All children with a primary diagnosis related to disturbances of the visual pathways (ICD-10 codes H47.619, H47.9 and ICD-9 codes 369.9, 377.75) aged 0–18 years presenting to a specialized neurology and neuro-ophthalmology clinic at a large pediatric hospital between January 2010 and September 2019 were selected from the institution’s electronic medical records. Subject inclusion was determined by a neuro-ophthalmologist (VS) upon chart review, to ensure an existing diagnosis of CVI. Patients were excluded if the baseline diagnosis was not consistent with CVI, or if there was insufficient clinical data available for analysis. A manual chart review was then conducted, and relevant information extracted including perinatal history, epilepsy history, developmental history (e.g., developmental delay and cerebral palsy), presence of a genetic diagnosis, baseline neuroimaging (brain MRI), and baseline fundoscopy. Patient follow-up longitudinal data was then collected and included CVI grading score (see below), presence of epilepsy (and epileptic encephalopathy), seizure control, current seizure medications, and therapy received. Ophthalmic examination including visual acuity, visual behavior, visual field preference, and fundoscopic details were also included at each time point. Neuroimaging findings were categorized by two pediatric neurologists (AJG, KF), depending on the suspected pattern(s) of injury. Ophthalmic evaluation data were categorized by two of the researchers (VSS, AJG).

### Cerebral visual impairment grading score

Given the (1) lack of a standardized CVI screening and assessment method, (2) the retrospective nature of this study, and (3) integration of both neurology and neuro-ophthalmology medical record data, a custom CVI severity grading system was established. Overall visual function was graded: (0) no blink to light from a transilluminator; (1) blink to light; (2) fixate and follow; (3) objective vision, assessed via Teller Acuity Cards or optokinetic drum response (binocular testing with a manual striped drum rotated at 8–10 rpm); and (4) subjective visual acuity. Additional single points were given for positive visual field/preference noted on exam, response to kinetics/objects (other than OKN drum), light gazing, and color preference (up to 4 additional points altogether). The CVI grading score was totaled for each patient and grouped into three categories of Severe (0–2 points), Moderate (3–5 points), and Mild (6–8 points), with lower scores reflecting more impairment.

### Cerebral visual impairment therapy

This study also sought to determine whether particular interventions targeted at improving visual function in patients contributed toward an improvement in our CVI grading score. A total of four interventions were assessed, which included: (1) Early Childhood Intervention (ECI) visual therapy services to help children and their families learn daily environmental adaptations that can maximize a child’s functional vision; (2) physical and occupational therapy (PT/OT) which involved motor development exercises that incorporated visuospatial training; (3) refractive correction with eyeglasses prescribed by cycloplegic retinoscopy according to the preferred practice patterns by the American Academy of Ophthalmology (AAO, 2021); and (4) a combination of all therapies. Of note, subjects undergoing refractive correction followed with their preferred provider and were not monitored for compliance or changes in their refraction by this study. The treatment effect of these interventions was assessed longitudinally via the CVI grading score on a per-encounter basis.

### Statistical analysis

For descriptive analysis, categorical variables were reported as count and percentage. Chi-square test or Fisher’s exact test (for contingency tables with at least 20% expected frequency < 5) were used to test the associations between categorized CVI grade outcomes (mild, moderate, and severe) and categorical variables.

To assess the treatment effect of CVI interventions, a mixed-effects model was used to model the longitudinal CVI grade scores over time to account for the multiple measurements from each patient. Baseline characteristics were tested one at a time. Perinatal characteristics, history of cerebral palsy, genetic diagnosis, and abnormal fundoscopy findings were pre-specified as candidate confounders for the outcome. The confounders that were found to be significantly associated with CVI grade outcome (*p* < 0.05), and the longitudinal measured variables at each visit (age and presence of epilepsy) were then included in a multivariable mixed-effects model. The type of treatment was the main variable of interest and was kept in the model regardless of its significance. Those that reached a significance of *p* < 0.1 were retained in the final multivariable model. The treatments being separately evaluated were: (1) Therapy documented at each visit, (2) Therapy documented in any one visit throughout the follow-up period (patient-level variable), (3) PT/OT documented at each visit, and (4) Glasses/refractive error correction documented at each visit.

No adjustments for multiple testing were made due to the exploratory nature of the study. A 2-sided *p* value < 0.05 was used to determine the significance of variables in all analyses. All statistical testing was performed using SAS 9.4.

## Results

A total of 249 subjects spanning 779 patient visits were included for analysis from time of initial CVI diagnosis. A summary of patient age and average follow-up period is included in Table 1. The mean age at time-of-diagnosis was 18.8 ± 16.8 months (2–108 months). Analysis of associations between each clinical factor and CVI grade severity group at baseline are documented in Table 2.

**TABLE 1:** Summary of patient age and follow-up information.

	Summary statistics	CVI cohort
**Baseline age (month)**		
	N	249
	Min–Max	2.0–108.0
	Mean (SD)	18.8 (16.83)
	Median (Q1, Q3)	13.0 (7.0, 24.0)
**Age at last visit (month)**		
	*N*	249
	Min–Max	2.0–157.0
	Mean (SD)	40.0 (24.75)
	Median (Q1, Q3)	35.0 (22.0, 54.0)
**Follow-up time (month)**		
	N	249
	Min-Max	0.0-84.0
	Mean (SD)	21.2 (20.71)
	Median (Q1, Q3)	15.0 (3.0, 33.0)
**Number of follow-up visits**		
	*N*	249
	Min–Max	1.0–10.0
	Mean (SD)	3.2 (2.05)
	Median (Q1, Q3)	3.0 (2.0, 4.0)

SD, standard deviation; Q1, 25th percentile; Q3, 75th percentile.

**TABLE 2:** Associations between each predictor and the CVI group at diagnosis.

Baseline characteristics	YES/NO	Moderate CVI (*N* = 52)	Severe CVI (*N* = 197)	Overall (*N* = 249)	*p* Value
**Abnormal perinatal history**					
Prematurity	Yes	32 (61.5)	128 (65.0)	160 (64.3)	0.647
	No	20 (38.5)	69 (35.0)	89 (35.7)	
Hypoxic-ischemic encephalopathy (HIE)	Yes	45 (86.5)	163 (82.7)	208 (83.5)	0.503
	No	7 (13.5)	34 (17.3)	41 (16.5)	
Intraventricular hemorrhage	Yes	45 (86.5)	175 (88.8)	220 (88.4)	0.652
	No	7 (13.5)	22 (11.2)	29 (11.6)	
Neonatal Seizures	Yes	39 (75.0)	156 (79.2)	195 (78.3)	0.520
	No	13 (25.0)	41 (20.8)	54 (21.7)	
Retinopathy of prematurity	Yes	48 (92.3)	182 (92.4)	230 (92.4)	1.000
	No	4 (7.7)	15 (7.6)	19 (7.6)	
**Epilepsy**					
Epilepsy	Yes	13 (25.0)	40 (20.4)	53 (21.4)	0.479
	No	39 (75.0)	156 (79.6)	195 (78.6)	
Epileptic encephalopathy	Yes	26 (66.7)	103 (66.0)	129 (66.2)	0.940
	No	13 (33.3)	53 (34.0)	66 (33.8)	
Poor seizure control	Yes	7 (17.9)	40 (25.6)	47 (24.1)	0.303
	No	32 (82.1)	116 (74.4)	148 (75.9)	
Medications used for control	None	17 (43.6)	35 (22.4)	52 (26.7)	0.033
	1-2	18 (46.2)	101 (64.7)	119 (61.0)	
	3 or more	4 (10.3)	20 (12.8)	24 (12.3)	
**Genetics**					
Known Genetic diagnosis	Yes	39 (75.0)	143 (72.6)	182 (73.1)	0.726
	No	13 (25.0)	54 (27.4)	67 (26.9)	
**Neurodevelopment**					
Cerebral palsy	Yes	24 (46.2)	74 (37.9)	98 (39.7)	0.232
	No	28 (53.8)	121 (62.1)	149 (60.3)	
Developmental delay	Yes	6 (11.5)	32 (16.2)	38 (15.3)	0.388
	No	46 (88.5)	165 (83.8)	211 (84.7)	
**Neuroimaging**					
Any abnormality	Yes	2 (4.4)	11 (6.6)	13 (6.2)	0.740
	No	43 (95.6)	155 (93.4)	198 (93.8)	
Atrophy	Yes	30 (66.7)	125 (75.3)	155 (73.5)	0.253
	No	15 (33.3)	41 (24.7)	56 (26.5)	
Presumed genetic etiology	Yes	32 (71.1)	122 (73.5)	154 (73.0)	0.751
	No	13 (28.9)	44 (26.5)	57 (27.0)	
Presumed metabolic etiology	Yes	43 (95.6)	150 (90.4)	193 (91.5)	0.374
	No	2 (4.4)	16 (9.6)	18 (8.5)	
Vascular changes	Yes	34 (75.6)	130 (78.3)	164 (77.7)	0.696
	No	11 (24.4)	36 (21.7)	47 (22.3)	
Prematurity-related changes	Yes	35 (77.8)	131 (78.9)	166 (78.7)	0.869
	No	10 (22.2)	35 (21.1)	45 (21.3)	
Presumed infection-related changes	Yes	43 (95.6)	156 (94.0)	199 (94.3)	1.000
	No	2 (4.4)	10 (6.0)	12 (5.7)	
Non-neonatal HIE	Yes	36 (80.0)	131 (78.9)	167 (79.1)	0.873
	No	9 (20.0)	35 (21.1)	44 (20.9)	
Hydrocephalus (not atrophy-related)	Yes	36 (80.0)	147 (88.6)	183 (86.7)	0.150
	No	9 (20.0)	19 (11.4)	28 (13.3)	
**Fundoscopic exam**					
Any abnormal finding	Yes	41 (78.8)	155 (78.7)	196 (78.7)	0.979
	No	11 (21.2)	42 (21.3)	53 (21.3)	
**Therapy/interventions**					
Any Therapy	Yes	44 (84.6)	165 (83.8)	209 (83.9)	0.880
	No	8 (15.4)	32 (16.2)	40 (16.1)	

*Chi-square test or Fisher’s exact test (for contingency tables with at least 20% expected frequency < 5) was used.

### Baseline characteristics

#### Cerebral visual impairment grade and ophthalmologic exam

According to the baseline CVI grading score of the 249 subjects, 197 (79.1%) had severe CVI with an average grade of 1.08, and 52 (20.9%) had moderate CVI with an average grade of 3.25, whereas no patients had CVI grades that qualified for mild CVI in this cohort. Visual examination of the cohort revealed no blink-to-light (BTL) vision in 24.5% of patients, BTL vision in 34.5%, fixate and follow vision in 26.5%, and preserved objective vision in 14.4%. No patients were found to have intact subjective visual acuity. Fundus examination with dilated pupils demonstrated abnormalities in 20.4% of the cohort including optic nerve pallor (49/51) and chorioretinal scarring or lacunae (3/51). The optic nerve pallor was bilateral and moderate-severe in 49% (24/51) of these patients.

#### Perinatal history

About 35.7% (*n* = 89) of the study cohort had a history of preterm birth [defined by the World Health Organization as any birth before 37 completed weeks of gestation (Howson et al., 2013)]. Within this preterm group, 25.8% (23/89) had a history of interventricular hemorrhage (IVH) of any grade, followed by 23.6% (21/89) neonatal seizures, and 21.3% (19/89) hypoxic-ischemic encephalopathy (HIE). In addition, 21.3% (19/89) also had a history of retinopathy of prematurity, but only 3 of these subjects had abnormal fundus findings. Conversely, only 3.8% (6/160) of the study cohort born at term had a history of IVH, 20.6% (33/160) had neonatal seizures, and 14.4% (23/160) had a history of HIE. Neonatal seizures were found in 21.7% (54/249) of all subjects, with 94.4% (51/54) in this group eventually developing future epilepsy.

The CVI grade was most severe in the HIE group with a mean of 1.24, followed by the neonatal seizures group with a mean of 1.44. IVH had a CVI mean grade of 1.55, with 34.5% (10/29) having abnormal fundoscopic findings.

#### Neuroimaging

About 93.8% (198/211) of those who had neuroimaging had documented changes. Among these, 28% had a presumed genetic/structural/migrational anomaly/etiology, 28.3% had atrophic changes on MRI not inherently related to prematurity, and 23.7% had vascular changes including ischemic and/or hemorrhagic stroke and intracranial bleeding (excluding prematurity-related intraventricular hemorrhage). A total of 22.7% had presumed prematurity-related changes (e.g., IVH, periventricular leukomalacia). HIE outside of neonatal origin was observed in 22.2%. Metabolic and presumed infection related changes were present in 9.1 and 4.8%, respectively. Lastly, hydrocephalus, presumed unrelated to atrophy (i.e., not *ex-vacuo*) was observed in 11.2% of all cases. Notably, abnormal neuroimaging for presumed metabolic and genetic etiologies had a CVI mean grade of 1.06 and 1.11, respectively. Alternatively, neuroimaging suggestive of vascular etiologies had a mean CVI grade of 1.43 with 31.9% (15/47) having abnormal retinal fundoscopic findings.

#### Neurodevelopment and genetics

Within this cohort, 84.7% (211/249) had a diagnosis of global developmental delay, with 59.8% (149/249) also known to have a diagnosis of cerebral palsy (CP). Among all individuals, 26.9% (67/249) had a known genetic diagnosis with a mean CVI grade of 1.54, of which 14.9% (10/67) had documented chromosomal abnormalities. Genetic testing for these individuals were originally requested for working up comorbid conditions (developmental delay, epilepsy) unrelated to CVI. A summary of identified gene and chromosomal abnormalities associated with this CVI cohort are listed in Table 3. 76.1% (51/67) of all subjects with known abnormal genetic testing had abnormal neuroimaging. Only 11.9% (8/67) had abnormal fundus exam findings.

**TABLE 3:** List of abnormal genes and/or chromosome rearrangements identified in the CVI cohort.

Genes identified	*ASPA (2), ATP7A, ATP1A3, CACNA1A, CDKL5 (3), CDG, COXPD11, DNM-1, EIF2B5, FLAD1, FOXG1 (2), G6PD, GNAO1, GRIN2B, HECW2, KIF1A, LIS1 (5), LypopylTrans1, MCAD, MECP2, MDS, OTC, PropACID, PURA, N2, RARS, SCN2A, SDHA, SLC1A4, SLC35A2, SCN1A/SPTAN1/SYN1, SMAD4, STXBP1 (2), TBCK, TSC TREX1 (3), T18, T21 (3), TUBA1A, ZNF630*

Chromosome abnormalities	Del Xp22, del Xp11.23, Gain 15q13.2q13.3, 5q del, del 10q11.22q11.23, Gain chr13, 4p- deletion, del:1q43, 9q34del, microdup 16p11.2 loss 18p22

#### Epilepsy and seizure control

A total of 78.6% (195/249) of the subjects had epilepsy, of which 75.9% (148/195) had suboptimal or poor seizure control, defined as having more than one seizure per month over at least 6 months (Chawla et al., 2002). 33.8% (66/195) had epileptic encephalopathy. The overall mean CVI grade for epilepsy was 1.46, which dropped to 1.19 with uncontrolled epilepsy. There was a statistically significant association between CVI grade and number of epilepsy medications in use at time-of-diagnosis (*p* = 0.033). Patients in the severe CVI group tended to be on more epilepsy medications than the moderate CVI group (64.7 vs. 46.2% on 1–2 meds; 12.8 vs. 10.3% on 3+ meds) (Table 2).

#### Longitudinal cerebral visual impairment grade outcome

To assess the effect of individual baseline characteristics on longitudinal CVI grade over time, we utilized linear mixed-effect models. Data from a combined total of 779 patient visits (including initial evaluation) from the 249 subjects were included. The contribution from individual baseline characteristics to longitudinal CVI grade is shown in Table 4, where a perinatal history of HIE (*p* = 0.043), cerebral palsy (*p* = 0.029), presumed metabolic etiology on neuroimaging (*p* = 0.049), and abnormal fundoscopy (*p* = 0.023) were found to be significantly associated with the longitudinally measured CVI grade outcome. Having a history of epilepsy (*p* = 0.051) and currently using multiple (≥3) medications for seizure control (*p* = 0.053) are near-significantly associated with longitudinal CVI grade. Conversely, good seizure control (*p* = 0.11) and hydrocephalus findings on neuroimaging that are not atrophy related (*p* = 0.104) demonstrate a trending association with longitudinal CVI grade improvement.

**TABLE 4:** Effect of individual baseline characteristics on longitudinal CVI grade outcome using mixed-effect models.

Baseline characteristics	Contrast	*N*	Parameter estimate (95% CI)	*p* Value
**Abnormal perinatal history**				
Prematurity	Yes vs. No	783	0.097 (-0.18, 0.37)	0.49
Hypoxic-ischemic encephalopathy (HIE)	Yes vs. No	783	-0.37 (-0.72, -0.012)	0.043
Intraventricular hemorrhage	Yes vs. No	783	-0.19 (-0.59, 0.21)	0.35
Neonatal Seizures	Yes vs. No	783	-0.26 (-0.58, 0.059)	0.11
Retinopathy of prematurity	Yes vs. No	783	-0.17 (-0.65, 0.32)	0.50
**Epilepsy**				
Epilepsy	Yes vs. No	782	-0.32 (-0.64, 0.0017)	0.051
Seizure control	Yes vs. No	606	0.29 (-0.065, 0.65)	0.11
Medications used for control	1–2 vs. zero	619	-0.33 (-0.66, 0.0037)	0.053
	>= 3 vs. zero	619	-0.57 (-1.10, -0.048)	
**Genetics**				
Known Genetic diagnosis	Yes vs. No	783	-0.031 (-0.33, 0.27)	0.84
**Neurodevelopment**				
Cerebral palsy (CP)	Yes vs. No	781	-0.30 (-0.57, -0.031)	0.029
Developmental delay	Yes vs. No	783	0.15 (-0.22, 0.51)	0.43
**Neuroimaging**				
Any abnormality	Yes vs. No	663	-0.024 (-0.62, 0.57)	0.94
Atrophy	Yes vs. No	663	0.0038 (-0.32, 0.33)	0.98
Presumed genetic etiology	Yes vs. No	663	0.077 (-0.24, 0.39)	0.63
Presumed metabolic etiology	Yes vs. No	663	-0.52 (-1.04, -0.002)	0.049
Vascular changes	Yes vs. No	663	-0.098 (-0.44, 0.24)	0.57
Prematurity-related changes	Yes vs. No	663	-0.27 (-0.61, 0.070)	0.12
Presumed infection-related changes	Yes vs. No	663	-0.078 (-0.70, 0.54)	0.81
Non-neonatal HIE	Yes vs. No	663	-0.24 (-0.59, 0.11)	0.18
Hydrocephalus (not atrophy-related)	Yes vs. No	663	0.34 (-0.069, 0.74)	0.104
**Fundoscopy**				
Any abnormal finding	Yes vs. No	783	-0.37 (-0.70, -0.053)	0.023

#### Effect of cerebral visual impairment therapy

Assessment of the effectiveness of CVI therapy was performed via multivariable mixed-effects models using longitudinal patient data from 779 patient visits. Upon including all significant baseline characteristics (Fundus abnormality, history of cerebral palsy, perinatal history of HIE) with longitudinal variables (age, presence of epilepsy, treatment received), the HIE variable lost its significance and was excluded. The final multivariate models, each focused on a particular therapy modality, are listed in Table 5. Overall, receiving some form of CVI therapy at each patient visit is significantly associated with an increased CVI grade outcome compared to no therapy (*p* = 0.043). For each patient, having at least one therapy recorded during their follow-up period is likely associated with an improved CVI grade compared to patients who never had any therapy recorded (*p* = 0.0646). PT/OT therapy was not significantly associated with improved CVI grade over time (*p* = 0.2468). However, pursuing refractive error correction with glasses and wearing them is associated with an average CVI grade increase of 0.21 units compared to those who did not wear glasses (*p* = 0.0363). Getting older (increased age) has a small but significant positive effect on CVI grade outcome regardless of therapy (0.01 unit increase in CVI grade for every month increase in age, or equivalently 0.12 unit increase in CVI grade for every year increase in age). Meanwhile, having active seizures, a positive history of cerebral palsy, and abnormal fundoscopic findings were all significantly associated with decreased CVI grade over time. The contribution of seizures is the largest out of the three, where patients with active seizures had on average a CVI grade of 0.4 points lower than patients who were not experiencing uncontrolled seizures.

**TABLE 5:** Treatment effect on Longitudinal CVI grade outcome using mixed-effect models.

Longitudinal analysis				
Treatment	*N*	Predictor variable	Estimate (95% CL)	*p*-Value
Therapy documented at each visit	779	Age	0.0100 (0.0066, 0.013)	<0.0001
		History of Epilepsy (Yes vs. No)	-0.38 (-0.58, -0.18)	0.0002
		Cerebral Palsy (Yes vs. No)	-0.35 (-0.61, -0.086)	0.0095
		Fundus Abnormality (Yes vs. No)	-0.32 (-0.63, -0.010)	0.0434
		Therapy At Each Visit (Yes vs. No)	0.14 (0.0044, 0.28)	0.0432
Therapy documented in any one visit throughout the follow-up period	779	Age	0.010 (0.0066, 0.013)	<0.0001
		History of Epilepsy (Yes vs. No)	-0.39 (-0.59, -0.19)	0.0001
		Cerebral Palsy (Yes vs. No)	-0.36 (-0.62, -0.096)	0.0076
		Fundus Abnormality (Yes vs. No)	-0.31 (-0.62, 0.0031)	0.0523
		Therapy At Any Visit (Yes vs. No)	0.25 (-0.015, 0.52)	0.0646
PT/OT documented at each visit	779	Age	0.010 (0.0067, 0.013)	<0.0001
		History of Epilepsy (Yes vs. No)	-0.38 (-0.58, -0.18)	0.0002
		Cerebral Palsy (Yes vs. No)	-0.35 (-0.61, -0.081)	0.0106
		Fundus Abnormality (Yes vs. No)	-0.32 (-0.63, -0.008)	0.0441
		PT/OT (Yes vs. No)	0.083 (-0.058, 0.22)	0.2468
Glasses documented at each visit	779	Age	0.0094 (0.0060, 0.013)	<0.0001
		History of Epilepsy (Yes vs. No)	-0.38 (-0.58, -0.18)	0.0002
		Cerebral Palsy (Yes vs. No)	-0.33 (-0.59, -0.069)	0.0133
		Fundus Abnormality (Yes vs. No)	-0.34 (-0.65, -0.031)	0.0311
		Glasses (Yes vs. No)	0.21 (0.013, 0.40)	0.0363

## Discussion

This study examined a large cohort of pediatric patients with a primary diagnosis of CVI with the goal of identifying neurologic, developmental, genetic, and neuro-ophthalmic predictors of CVI severity and longitudinal treatment response to vision-based interventions.

### Ophthalmologic exam

It is well known that CVI impacts vision on a clinical spectrum of mild visual disturbances to profound vision dysfunction. Despite devising a metric-based CVI grading system aimed at classifying mild, moderate, and severe instances of vision impairment, we found that no pediatric patients in our cohort met clinical criteria in their baseline ophthalmic examinations to be designated as having mild CVI. Rather, our cohort only describes moderate and severe cases of CVI and may reflect the fact that our larger patient population represents a tertiary referral pool with a skew toward having more severe underlying conditions and comorbidities requiring subspecialty evaluation. Conversely, pediatric patients who meet criteria for mild CVI may present with high-order visuospatial impairment that are underdiagnosed or unrecognized for appropriate workup, diagnosis, and referral (van Genderen et al., 2012; Chandna et al., 2021).

Despite not capturing the milder clinical spectrum of CVI, our scoring system helped stratify moderate from severe disease. Additionally, more than half of our cohort had severely decreased vision evident on visual function testing with 23.3% (*n* = 58) demonstrating an absent BTL response and 34.5% (*n* = 86) with only a BTL response and no further visual capability. However, CVI can also present with additional ocular comorbidities including strabismus, ocular dysmotility, and optic nerve/retinal changes. Although we did not assess for strabismus due to the variability of the documented exam, the dilated fundus exam detected optic nerve abnormalities and chorioretinal lesions (scarring or lacunae) in our cohort. Optic nerve pallor was found in 17% (51/249) of our cohort, was noted to always present bilaterally, and ranged from isolated temporal to diffuse pallor. The presence of optic nerve pallor in these CVI children could result from either co-morbid anterior visual pathway dysfunction (e.g., congenital optic nerve abnormalities) or retrochiasmal pathology involving the optic radiation (e.g., periventricular leukomalacia) and resultant trans-synaptic degeneration (Dutton, 2003; Hoyt, 2003; Good, 2007; Lennartsson et al., 2018). Overall, our ophthalmic findings in this study are consistent with prior works that reported the prevalence of optic nerve pallor (16–42%) in CVI children (Huo et al., 1999; Hoyt, 2003; Khetpal and Donahue, 2007; Handa et al., 2018).

### Perinatal and neuroimaging findings

In the 160 children of our cohort born at term, 21.3% presented with neonatal seizures, 13.1% with HIE and 3.1% with IVH. Prior studies have reported that HIE is the most common cause of CVI in preterm and term children (Good et al., 1994; Huo et al., 1999; Fazzi et al., 2007; Khetpal and Donahue, 2007). Interestingly, our cohort reported a higher prevalence of neonatal seizures than HIE in both term and preterm children as an etiology of CVI. In addition, the incidence of neonatal seizures (*n* = 54) in the perinatal period in both preterm and term children had a higher propensity for developing into epilepsy (*n* = 51/54), and to a lesser degree, epileptic encephalopathy (*n* = 16/54). This observation suggests that neonatal seizures may be a potential prognostic sign for a systemic disease course and raises concerns for potential development of CVI.

The distinction of prematurity, defined as a child born before 37 completed weeks of gestation, is a critical feature of characterizing CVI in children. With 35.7% (*n* = 89) of our cohort meeting criteria for preterm birth, neurologic clinical assessment (clinical history, APGAR score, blood gas, Sarnat staging—classification scale for HIE) and/or initial imaging demonstrated co-morbid pathologic findings consistent with IVH (25.6%; *n* = 23/89), neonatal seizures (23.6%; *n* = 21/89), and HIE (21.3%; *n* = 19/89). As IVH and HIE are potential contributing precursors of periventricular leukomalacia (PVL), our findings are consistent with prior studies describing an association with preterm babies presenting with PVL on neuroimaging (Banker and Larroche, 1962; Jacobson and Dutton, 2000; Dutton, 2013). Finally, retinopathy of prematurity (ROP) was found in 21.3% (*n* = 19/89) of our cohort. Per medical record review, these children were monitored postnatally but none experienced any vision threatening sequalae.

While neuroimaging alone cannot diagnose CVI, it does provide valuable anatomical insights into developmental etiologies. A strength in our study was the fact that a majority of patients (211/249) had existing neuroimaging at the time of their initial ophthalmologic evaluation, and a vast majority had abnormal findings. Neuroimaging in CVI can reveal a spectrum of pathology ranging from focal to global brain involvement (Whiting et al., 1985; Eken et al., 1996; Ortibus et al., 2009). In our cohort, 93.8% presented with an abnormal finding on neuroimaging, the most common being structural anomalies suggestive of an underlying genetic etiology (e.g., migrational abnormalities) in 28.8%, followed closely by atrophy (unspecified cause, 28.2%). Outright presumed sequelae of prematurity (e.g., periventricular leukomalacia), and vascular insults in any location were also frequently reported, 22.7 and 23.7%, respectively (Bauer and Papadelis, 2019). In our study, no association was found between the type and location of identified neuroimaging findings on MRI, and the degree/severity of CVI at time-of-diagnosis, consistent with prior findings (Cioni et al., 1996; Chang and Borchert, 2020). This would suggest that, while structural neurologic injury plays a role in the pathogenesis of CVI, there may be other functional tests and characteristics of neuroimaging that better account for the severity of CVI and perhaps warrant more attention than baseline imaging (Fazzi et al., 2009; Ortibus et al., 2009). This observation is consistent with the notion that standard neuroimaging modalities are limited in their capability to correlate visual function with neuroanatomical changes in space and time. However, recent developments in novel techniques such diffusion tensor imaging and high angular resolution diffusion imaging (HARDI) will allow future investigators to closely examine white matter connectivity in the context of dysfunction and better elucidate potential pathways involved in CVI (Ortibus et al., 2009; Bauer et al., 2014; Martín et al., 2016).

### Neurodevelopment and genetics

A striking majority of our CVI patients presented with either some form of developmental delay (DD) or cerebral palsy (CP) (231/249, 93.1%). We also found in our longitudinal cohort that CP at initial diagnosis was significantly associated with worsening CVI grade outcome over time. This is suspected to be due to multiple reasons. Both DD and CP are clinical diagnoses that may be comorbid, and children with CVI commonly have more than one underlying neurologic disorder (Castano et al., 2000; Fazzi et al., 2007; Handa et al., 2018). Additionally, DD and CP represent a large group of heterogeneous conditions that not only have highly variable etiologies, but in fact share many of them with CVI (e.g., prematurity, developmental and epileptic encephalopathies, and known genetic conditions).

As many as 26.1% of our patient cohort had a formal genetic diagnosis, including large chromosomal abnormalities (trisomy, deletions, duplications) and single gene pathogenic variants. The extent of our cohort’s abnormal genetic findings exceeded that of prior studies (Matsuba and Jan, 2006; Bosch et al., 2014). Despite this, we still believe this underestimates the true incidence of genetic anomalies among children with CVI. Most of our genetic testing was obtained through a separate neurology clinic, where the focus was on other comorbid neurologic conditions (CP, DD, epilepsy), rather than CVI. Instead, a dedicated rigorous approach in genetic testing has the potential to uncover a larger number of etiologic targets. Our study nonetheless identified genes that have previously been implicated in CVI pathogenesis (*CDKL5, SLC35A2, LIS1*) (Bosch et al., 2014), as well as those not described elsewhere in the literature (*COXPD11, EIF2B5, FLAD1*). Despite these findings however, our study ultimately did not reveal a significant correlation between CVI severity/improvement and underlying genetic diagnosis. Instead, a rigorous and unbiased approach that examines a more homogeneous patient population (i.e., no neurological comorbidities) may be valuable.

### Epilepsy

Most individuals of our cohort had a diagnosis of epilepsy at time of initial ophthalmologic evaluation (78.3%, *n* = 195/249). Among these, 33.8% had an identified form of epileptic encephalopathy (cerebral dysfunction related to often difficult-to-control epileptic activity), with infantile spasms/hypsarrhythmia being most common (83.3% of said group). This is likely related to the timing of initial ophthalmologic diagnosis vis-à-vis the most recent electroencephalographic studies. Nonetheless, these clinical findings are consistent with prior reports that have noted a significant seizure component in pediatric CVI patients, the majority of which were infantile spasms (Huo et al., 1999; Khetpal and Donahue, 2007; Handa et al., 2018).

With regards to seizure control, there is contradictory evidence in which earlier studies have reported improved visual development with improved seizure control, and a correspondingly poor prognosis for children with uncontrolled seizures (Wong, 1991; Good et al., 1994). However, it has also been recently reported that seizure control did not significantly improve vision, as well as evidence that seizure may not be contributory to CVI (Grant et al., 2008; Handa et al., 2018).

In the epilepsy group, 75% of patients had optimal seizure control at time-of-diagnosis of CVI. The mean CVI grade was more severe for patients with uncontrolled epilepsy (1.19) than controlled epilepsy (1.54). A trend toward mean CVI grade improvement was seen with improved seizure control. Interestingly, the use of multiple antiseizure medications (3 or more antiseizure medications) correlated with an increased baseline severity of CVI (Table 2). This may be indicative of the severity of the underlying epilepsy syndrome, and possibly a more reliable marker of epilepsy disease burden than subjective reporting of optimal or poor control. Additionally, the use of multiple antiseizure medications was also negatively associated with longitudinal CVI outcome, and suggests that seizure activity reflects a cumulative burden on CVI prognosis over time. Of additional interest, a number of patients with a history of infantile spasms or other early epilepsy syndromes may have received medications known to impair visual function (e.g., vigabatrin); however, given the lack of usual related findings (e.g., visual field loss), this may not sufficiently explain the difference in CVI grade across groups. Practically, it is important for the vision care provider to consider the number of seizure medications and whether if it may be affecting the child’s performance during a CVI vision assessment. This would importantly pair up with the increasing tendency toward early aggressive seizure/epilepsy management among neurologists, including offering earlier curative or palliative epilepsy surgery options and few medication trials (Prideaux et al., 2018; Roth et al., 2021; Perry et al., 2022).

### Therapy/interventions

Presently, there is no standardized treatment for CVI. Given its heterogeneous nature and the broad range of visual impairment, it has been difficult to design and execute studies determining if a particular therapy or intervention is effective. Additionally, the paucity of evidence on the clinical effectiveness of CVI-based therapy makes our study unique in that our cohort was longitudinally followed for treatment effect after receiving multimodal interventions. It has been previously reported that improvements in vision in CVI patients were not associable to any unique etiology (Huo et al., 1999; Handa et al., 2018). Accordingly, our study cohort received therapies targeted at CVI visual improvement, including ECI vision therapy, visual rehabilitation (PT/OT), refractive error correction, and combination therapy.

Out of the therapy modalities that were offered to patients, we found that refractive correction with glasses was associated with the greatest improvement in CVI grade outcome over time. This is compared to ECI, PT/OT, or a combination of all therapies, which did not demonstrate significance with longitudinal CVI grade. Correcting refractive error is pursued for multiple pediatric conditions, and our study provides evidence that it is viable therapy for moderate and severe pediatric CVI as well. Prior studies have also identified treatable associated ophthalmic conditions (e.g., refractive error, accommodative insufficiency, cataract) in a significant proportion of CVI patients (Philip and Dutton, 2014; Pehere et al., 2018). Taken together, our evidence suggests that patients ought to pursue management for treatable ophthalmic conditions that co-present with CVI.

As an aggregate, having therapy documented at any visit was strongly associated with an average improvement in CVI grade of 0.14 per month, compared to no therapy. This was additionally supported at the patient level, whereby having at least one therapy recorded at any visit during a patient’s follow-up period was also associated with a mean improvement in CVI grade of 0.25 per month. While refractive error correction as mentioned above may represent the majority contribution toward this improvement, the natural development of the visual system in pediatric patients may also play a role. In our multivariate model, age played a small but significant positive contribution toward CVI grade outcome, but the overall effect size is insufficiently large to explain the total improvement afforded by receiving CVI therapy during follow-up.

### Limitations

Our study has multiple limitations that are common in retrospective studies. The CVI grading system described in this study was an attempt to incorporate subjective and objective clinical data, but may not have encompassed a true and robust quantification of visual function and behavior. A grading system developed after patient data collection may help better refine our CVI scale for future prospective studies. Given the retrospective nature of this study, certain aspects of data collection may be incomplete (information bias). Particularly, our data from genetic testing arose from workup targeted at separate underlying conditions (e.g., epilepsy), and may not represent the optimal context to identify causative genes associated with CVI. Due to the retrospective nature of the study, therapy modalities were not implemented in an evenly distributed manner. Additionally, our unbalanced sample sizes for our moderate vs. severe CVI groups (*n* = 52 vs. 197) reduces the statistical power.

## Conclusion

The diagnosis and management of CVI is challenging due to its variable clinical manifestations, the presence of additional comorbidities, and the myriad associations it shares with other conditions. Herein, we present an extensive analysis of a retrospective cohort of pediatric patients with advanced CVI, showing that (1) perinatal HIE history, abnormal fundoscopic findings, and cerebral palsy are all negative predictors of CVI improvement; (2) optimizing seizure medications and epilepsy control served to benefit CVI improvement; and (3) patients who underwent longitudinal therapy, in particular refractive error correction with glasses demonstrated CVI score improvement from baseline and in comparison to an untreated cohort. This study also adds to the limited knowledge of genetics in CVI, and also provides a conceptual framework for the medical provider to screen for disease burden in the context of accessible visual function testing. Given that our approach reveals associations consistent with previously reported comorbidities (HIE, epilepsy) that correlate with CVI, future work that evaluates the relationship between visual function development and the etiological overlap shared by these comorbidities can enhance our understanding of CVI pathogenesis, with the goal of identifying avenues of therapy and support for affected children.

## Data availability statement

The raw data supporting the conclusions of this article will be made available by the authors, without undue reservation.

## Ethics statement

The studies involving human participants were reviewed and approved by Institutional Review Board Baylor College of Medicine. Written informed consent from the participants’ legal guardian/next of kin was not required to participate in this study in accordance with the national legislation and the institutional requirements.

## Author contributions

AJ-G, KF, and VS participated in study design, data collection and analysis, and manuscript draft and editing. CL and QS participated in data collection and analysis and manuscript draft and editing. KZ contributed to the statement. All authors contributed to the article and approved the submitted version.

## Conflict of interest

The authors declare that the research was conducted in the absence of any commercial or financial relationships that could be construed as a potential conflict of interest.

## Publisher’s note

All claims expressed in this article are solely those of the authors and do not necessarily represent those of their affiliated organizations, or those of the publisher, the editors and the reviewers. Any product that may be evaluated in this article, or claim that may be made by its manufacturer, is not guaranteed or endorsed by the publisher.
